# Development of Tiny Vane-Type Magnetorheological Brake Considering Quality Function Deployment

**DOI:** 10.3390/mi14010026

**Published:** 2022-12-22

**Authors:** Agus Lutanto, U Ubaidillah, Fitrian Imaduddin, Seung-Bok Choi, Bhre Wangsa Lenggana

**Affiliations:** 1 Department of Mechanical Engineering, Faculty of Engineering, Universitas Sebelas Maret, Surakarta 57126, Indonesia; 2 Department of Mechanical Engineering, Islamic University of Madinah, Medina 42351, Saudi Arabia; 3 Department of Mechanical Engineering, The State University of New York, Korea (SUNY Korea), Incheon 21985, Republic of Korea; 4 Department of Mechanical Engineering, Industrial University of Ho Minh City (IUH), Ho Chi Minh City 70000, Vietnam

**Keywords:** magnetorheological fluid, vane magnetorheological brake, tiny magnetorheological brake, ankle foot orthosis, quality function deployment

## Abstract

Many studies focus on the torque-to-dimension ratio when designing magnetorheological brakes (MRB), especially for ankle foot orthosis (AFO) devices. Vane MRB is one type of MRB with a limited angle of motion that is naturally suitable to be applied to AFO. However, very few implement quality function deployment (QFD) when making MRB, whereas QFD is an essential factor in making product designs. In this study, a tiny vane-type MR brake (TVMRB) was successfully made using the QFD method. Torque characteristics are determined by analysis of magnetic flux density, theoretically, by 3D simulation, and by using Ansys Maxwell experimentally. For consideration, the analysis was carried out with fluid gap variations (0.5 mm, 0.75 mm, and 1 mm) and current variations (0.5–2 A with 0.5 A increments). As a result, ignoring the leakage of MR fluid (MRF), at a constant rotation of 10 rpm, the smallest torque of 6.14 Nm was obtained at the fluid gap variation of 1 mm and input current of 0.5 A, whereas the largest torque was 46.71 Nm at the fluid gap variation of 0.5 mm and input current of 2 A. Apart from torque, this article will also discuss other brake performances in the form of operational range and power consumption. Finally, the structure of the TVMRB design is compared with other designs presented in the House of Quality (HOQ).

## 1. Introduction

Continuous improvement brings innovation in technology development, including in wearable rehabilitation devices. There are two types of wearable rehabilitation devices, namely prostheses and orthosis. Prostheses replace damaged or lost limbs such as legs and arms with nearly the same function. In comparison, an orthosis is a device to support a disturbing limb to be in the correct position [1,2,3]. For example, an orthosis is widely applied to the ankle to improve gait for stroke patients [4]. Ankle foot orthosis (AFO) consists of rigid and articulated types. The rigid type has mechanical capabilities that cannot be controlled, and the articulated type has more potential to be developed [5]. Articulated AFO uses several actuators, one of which is a magnetorheological brake (MRB). Magnetorheological fluid (MRF) is an intelligent material where when affected by a magnetic field, its viscosity will increase in milliseconds so that its shape becomes non-Newtonian. Meanwhile, when unaffected by a magnetic field, the MRF viscosity decreases and returns to Newtonian form [6,7]. The unique properties of the MRF are exploited to generate torque on the MRB. Torque can be adjusted by adjusting the strength of the magnetic field.

In general, there are three working modes of MRF, namely, shear mode [8,9,10], flow mode [11,12], and squeeze mode [13,14]. The shear mode applies the friction principle wherein the MRF is placed in the gap between two magnetic components, one moving and the other stationery. Flow mode, also known as valve mode, is the mode in which the MRF flows between two magnetic components. Poiseuille’s law applies in flow mode because when the MRF flows in the valve gap, there is a pressure difference on both sides [15]. Squeeze mode is a condition when the MRF is between two magnetic components that are compressed and decompressed under certain conditions. Of the three modes, shear mode and flow mode are the modes that are often used in MRB. However, the shear mode has a weakness; the end of the chain bond is the weakest point, so the external force of the moving magnetic component causes the bonds between particles to be easily damaged [16,17]. In addition to the MRB, pneumatic and hydraulic actuators were also developed. Li and Hsiao-Wecksler [18] made a portable AFO with power from compressed CO_2_ with a torque of up to 12 Nm. With the same type of actuator, Kim et al. [19] reduced the air compressor’s size and produced 9.8 Nm of torque. Neubauer and Durfee [20], with a total weight of 3.3 kg for a hydraulic ankle foot orthosis (HAFO) device, can produce 65 Nm of torque. However, unfortunately, although the pneumatic and hydraulic types can achieve high torque, both types still require a pump and reservoir to increase in size and weight. In addition, a connecting hose that runs across from the power supply attached to the patient’s body to the AFO with legs makes it difficult to operate. To minimize these shortcomings, the use of MRB can be an alternative.

MRB is divided into three types: linear, continuous, and vane. Linear MRB works by inhibiting translational motion, and continuous MRB works on objects that can rotate 360 degrees, whereas vane MRB can only move at a limited rotation angle. Oba et al. [10] made a shear mode viscosity link linear MRB combined with a compression spring. Hassan et al. [2] improved the design of Oba et al. [10] by reducing the fluid gap from 1 mm to 0.5 mm, changing the size of the bobbin core to be thicker to minimize the effect of saturation of the magnetic field, and increasing the number of coils. The result is that the torque produced reaches 11 Nm with a current of 1 A. Meanwhile, Furuso et al. [21], Kikuchi et al. [22], and Zhou and Liu [3] made a continuous MRB with a multi-disc array. Furuso et al. [21] developed a compact MRB, compiling seven static disks and six rotor disks with a gap of 50 μm. The resulting torque is 11.8 Nm, a good result in terms of torque-to-dimension ratio considering that the MRB is only 69 mm in diameter and 29 mm thick. The design was then made more compact by Kikuchi et al. [22], resulting in torque compensation of 5 Nm. Although it succeeded in making a compact MRB design with large torque, the manufacturing process is quite complicated because the gap between disks is very small. Zhou and Liu [3] created the new MRB configuration with a multi-layer arrangement and coils separated into two parts. However, in this study, no further discussion was made on the resulting torque. The MRB compact was also successfully developed by Ubaidillah et al. [4] and Hidayatullah et al. [8] with serpentine flux configuration. Despite having a compact design, the torque produced is small, which is 0.26 Nm and 2.1 Nm, respectively.

In contrast to linear and continuous types, vane MRB have rarely been implemented. Only a few designs were found for prosthetics [12,23], vehicle dampers [24,25,26], and back-drivable robotic industries [27]. None of these has been applied to AFO devices, whereas according to Rahman et al. [28], MRB vanes are capable of producing greater torque when compared to other types. From some literature, researchers are trying to design the MRB with a compact size and high torque. However, so far, there are very few studies that make scalable MRB designs based on quality function deployment (QFD). QFD is a critical design stage so that the product design follows customer needs. The design process will also be more effective by prioritizing priority factors [29].

Therefore, the novelty in this study is to design a potential MRB type for the AFO device, namely the tiny vane-type MRB (TVMRB), which has never been carried out before. Here, a new analysis method was also used to determine the best design parameters by combining brake performance results with QFD. This study does not discuss further clinical feasibility but rather the design requirements and analysis of toque characteristics based on magnetic flux analysis using theoretical, simulation, and experimental. Then, the HOQ will be shown as the basis for the feasibility of the design before it is produced.

## 2. Materials and Methods

### 2.1. Quality Function Deployment Concept

The QFD is a type of method for manufacturing products that prioritizes customer needs by considering the technical characteristics. The main focus is to involve users in the product design so that the product is appropriate and in great demand. The concept is to bring together customer requirements and technical requirements to determine the relationship between both [30]. By applying QFD, the priority scale of the design work will be known according to customer needs, the existence of a structured plan from making the design to the production process, and the ability to get an overview of product quality comparisons with other competing products [29].

**Figure 1:**
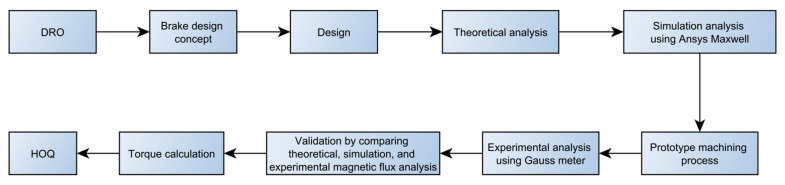
The flow chart for design and analysis of TVMRB.

The flow in this study follows the steps shown in Figure 1. Before making a design, it is necessary to determine the design requirements and objectives (DRO) which will be the focus of the research. First, the design concept was made by considering the angle of movement and size of the TVMRB. After that, the valve design is made, where the geometry of the valve will determine the electromagnetic circuit, fluid gap, and turn-ampere (multiplied by the number of turns of wire and current). These three factors are critical in the formation of torque. The design is then analyzed theoretically, through multi-objective design problem simulation, and experimentally on the prototype made. The best performance can be determined using an analysis carried out with fluid gaps and current variations. Finally, the analysis results are compared with other designs presented in the HOQ to determine the feasibility of the design.

### 2.2. Design Requirement and Objective 

**Figure 2:**
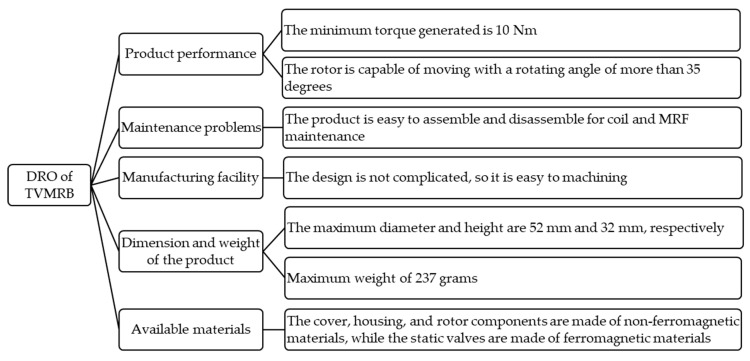
Design requirements of TVMRB and objective of the research.

As shown in Figure 2, the DRO contains the criteria TVMRB must have, so it becomes a reference in the design. The criteria are compiled by gathering information from the literature study on AFO needs. Then, it was determined that the DRO in this study contained product performance, ease of maintenance, ease of manufacture, compactness, and suitability of the materials used. TVMRB is targeted to achieve a minimum torque of 10 Nm to have a rehabilitative effect [18,22]. In addition, the angle of motion of the rotor refers to the ability to move the ankle, where the average movement is 70 degrees [31]. When walking, the ankle’s angle of motion is no more than 35 degrees [32]. The design is also made with a level of complexity, but it should be easy to be manufactured, assemble, and disassemble for maintenance, both coil and MRF. Besides torque and complexity, size and weight are essential in making the design because they will affect the comfort when installed on the patient’s foot. For this reason, in designing the MRB, the researchers put the compact design as the main parameter besides torque. TVMRB is targeted to have a more compact size than the MRB made by Kikuchi et al. [22], which has a maximum diameter of 52 mm, a height of 32 mm, and a weight of 237 g.

### 2.3. Structure Design and Materials

**Figure 3:**
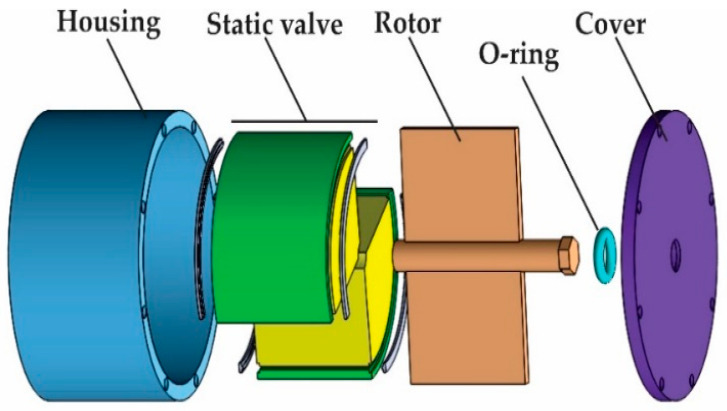
TVMRB design structure and components.

The structure of TVMRB is shown in Figure 3. There are four main components: cover, housing, rotor, and static valve. The cover, housing, and rotor are made of aluminum, whereas the static valve (inner and outer valve) is made of low carbon steel AISI 1010. The rotor blade and static valve are attached to the housing, where MRF will fill the remaining volume. AISI 1010 is a ferromagnetic material with a magnetic permeability of 667.75 $\mu_{0}$ H/m, where $\mu_{0}$ shows the magnetic permeability of the air with a value of 4π × 10^−7^ H/m [33], whereas the MRF used is the MRF-132 DG type from Lord Corporation, with specifications shown in Table 1.

**Figure 4:**
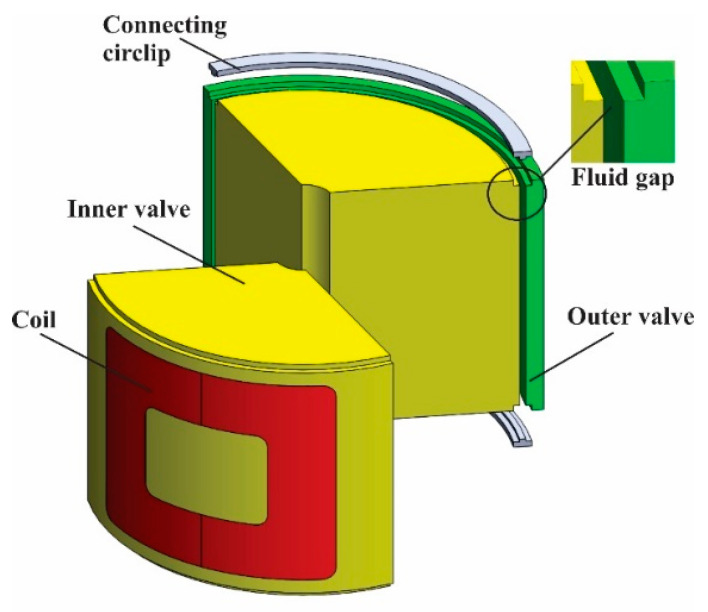
Static valve design configuration.

**Figure 5:**
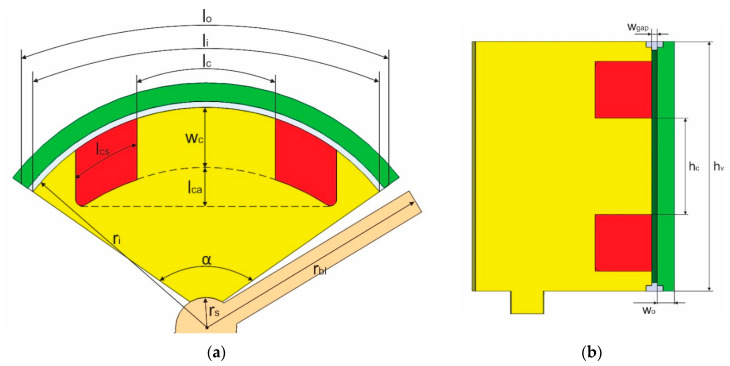
Dimensional specifications of the static valve: (**a**) Top view; (**b**) Side view.

The rotor blade configuration and static valve form two pairs of chambers connected by a fluid gap. The static valve design, as shown in Figure 4, consists of an inner valve, outer valve, connecting circlip, and a coil wrapped around the space in the inner valve. The material distribution of each component is shown in Table 2. When the static valve is not affected by a magnetic field, the rotor can move by 70 degrees. MRF can move from one chamber to another through the fluid gap. However, when the coil is energized, the MRF is affected by a magnetic field. There is a change in the MRF phase from Newtonian to Non-Newtonian; as a result, the rotor movement will be hampered, and braking torque will occur. Ignoring the leakage between the (i) rotor blade and housing, (ii) rotor shaft and inner valve, and (iii) rotor blade, static valve, and cover, the total torque can be calculated by Equation (1).

(1) $$T=2A_{bl}r_{bl}\Delta P_{t}$$

(2) $$\Delta P_{t}=\Delta P_{off-state}+\Delta P_{on-state}$$

where $A_{bl}$ is the blade surface area driving the MRF, $r_{bl}$ is the blade rotor radius, $\Delta P_{t}$ is the sum of the pressure drop when there is no magnetic field ($\Delta P_{off-state}$) and the pressure drop when the magnetic field is affected ($\Delta P_{on-state}$), as in Equation (2). $\Delta P_{off-state}$ and $\Delta P_{on-state}$ can be evaluated using Equations (3) and (4).

(3) $$\Delta P_{off-state}=\frac{12\eta QL}{h_{v}w_{gap}{}^{3}}$$

(4) $$\Delta P_{on-state}=\frac{3L\tau_{y}}{w_{gap}}\;$$

where η is the viscosity of the MRF-132 DG when off-state, Q is the flow rate, L is the length of the fluid gap, $h_{v}$ indicates the static valve height, $w_{gap}$ is the fluid gap, and $\tau_{y}$ indicates the yield stress of MRF-132 DG when on-state. As for the flow rate, Q can be determined by Equation (5).

(5) $$Q=\frac{1}{2}\omega \left(r_{bl}{}^{2}-r_{s}{}^{2}\right)h_{bl}$$

where ω is the angular speed of the rotor blade, $r_{s}$ is the radius of the rotor shaft, and $h_{bl}$ represents the height of the rotor blade. To help solve Equations (1)–(5), Figure 5 presents geometrical parameters and Table 3 gives the specific values of each design parameter. The design values are determined by considering the target dimension and weight of the DRO.

The design has variations in the fluid gap and current to determine the torque characteristics. The fluid gap and current parameters can significantly affect the torque produced. However, variations need to be limited. The fluid gap that is too small can inhibit the MRF rate when off-state, but if it is too large, it will reduce the total torque, whereas the current variation needs to be limited, considering the MRF has a saturation level. A greater current can also lead to high-temperature coils, which can reduce the performance of TVMRB [35,36]. In this study, the fluid gap variations given were 0.5 mm, 0.75 mm, and 1 mm, with a current variation of 0.5 A, 1 A, 1.5 A, and 2 A.

### 2.4. Electromagnetic Circuit Model

The magnetic flux in the fluid gap area is the key to the emergence of torque on the TVMRB. Determining the magnetic flux density can be formulated as follows, as seen in Equations (6) and (7):

(6) $$F=NI=B_{gap}A_{gap}R$$

(7) $$B_{gap}=\frac{NI}{A_{gap}R}$$

With F as the magnetic force, NI indicates the turn-ampere, $B_{gap}$ is the magnetic flux density in the fluid gap area, $A_{gap}$ is the surface area of the fluid gap affected by the magnetic field, and R is the magnetic resistance or called reluctance. The amount of reluctance can be expressed in Equation (8).

(8) $$R=\frac{L_{c}}{\mu A}$$

Here, $L_{c}$ is the magnetic flux circuit’s length, μ is the material’s permeability, and A is the surface area of the circuit.

**Figure 6:**
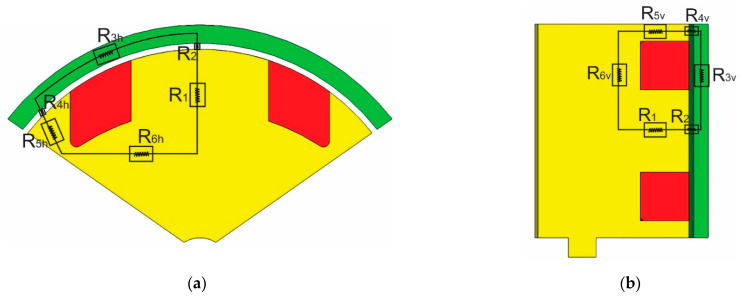
Electromagnetic circuit schematic: (**a**) Horizontal direction; (**b**) Vertical direction.

TVMRB has two electromagnetic circuit models, as shown in Figure 6. When the coil is energized, a magnetic flux appears with a close loop flow through the reluctance of the coil core ($R_{1}$)—fluid gap ($R_{2}$)—outer valve ($R_{3}$)—fluid gap ($R_{4}$)—inner valve ($R_{5}$ and $R_{6}$)—then returns to the coil core. In the outer coil, the magnetic flux is divided into horizontal and vertical directions, as shown in Figure 6a,b. By applying Equation (8), each reluctance value can be expressed into Equations (9)–(18).

(9) $$R_{1}=\frac{w_{c}+l_{ca}}{\mu_{st}h_{c}l_{c}}$$

(10) $$R_{2}=\frac{w_{gap}}{\mu_{MRF}h_{c}l_{c}}$$

The calculation of reluctance that moves horizontally $(R_{3h}$–$R_{6h}$) can be written into Equations (11)–(14).

(11) $$R_{3h}=\frac{l_{o}}{2\mu_{st}h_{v}w_{o}}$$

(12) $$R_{4h}=\frac{2w_{gap}}{\mu_{MRF}h_{v}\left(l_{i}-l_{c}-2l_{cs}\right)}$$

(13) $$R_{5h}=\frac{w_{c}}{\mu_{st}h_{v}\left(l_{i}-l_{c}-2l_{cs}\right)}$$

(14) $$R_{6h}=\left(\frac{\alpha }{360}\right)\;\frac{lnln\;\left[\frac{\left(r_{i}-w_{c}\right)}{r_{s}}\right]\;}{\mu_{st}\pi h_{v}}$$

Whereas the calculation of reluctance that moves vertically $(R_{3v}$–$R_{6v}$) can be written into Equations (15)–(18).

(15) $$R_{3v}=\frac{h_{v}}{2\mu_{st}l_{o}w_{o}}$$

(16) $$R_{4v}=\frac{2w_{gap}}{\mu_{MRF}l_{i}\left(h_{v}-h_{c}-2l_{cs}\right)}$$

(17) $$R_{5v}=\left(\frac{\alpha }{360}\right)\;\frac{lnln\;\left[\frac{r_{i}}{\left(r_{i}-w_{c}\right)}\right]\;}{\mu_{st}\pi \left(h_{v}-h_{c}-2l_{cs}\right)}$$

(18) $$R_{6v}=\frac{h_{v}}{(\frac{\alpha }{360})2\mu_{st}\pi [\left(r_{i}-w_{c}{)}^{2}-r_{s}{}^{2}\right]}$$

**Figure 7:**
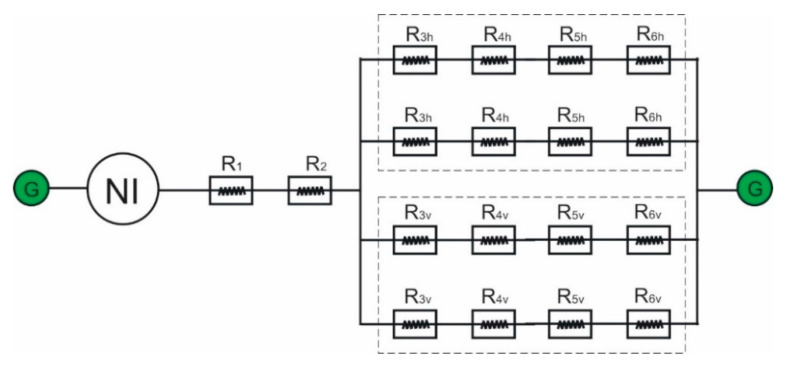
Complete circuit form of the electromagnetic circuit.

The complete TVMRB electromagnetic circuit can be described as shown in Figure 7. In the outer valve, two horizontal reluctances and two vertical reluctances are formed in parallel. The totals of both are arranged in series with $R_{1}$ and $R_{2}$. Therefore, the total reluctance can be expressed by Equation (19).

(19) $$R_{total}=R_{1}+R_{2}+{\left[\;\frac{2}{(R_{3h}+R_{4h}+R_{5h}+R_{6h})\;}+\frac{2}{(R_{3v}+R_{4v}+R_{5v}+R_{6v})\;}\right]}^{-1}$$

### 2.5. Testing Scheme

**Figure 8:**
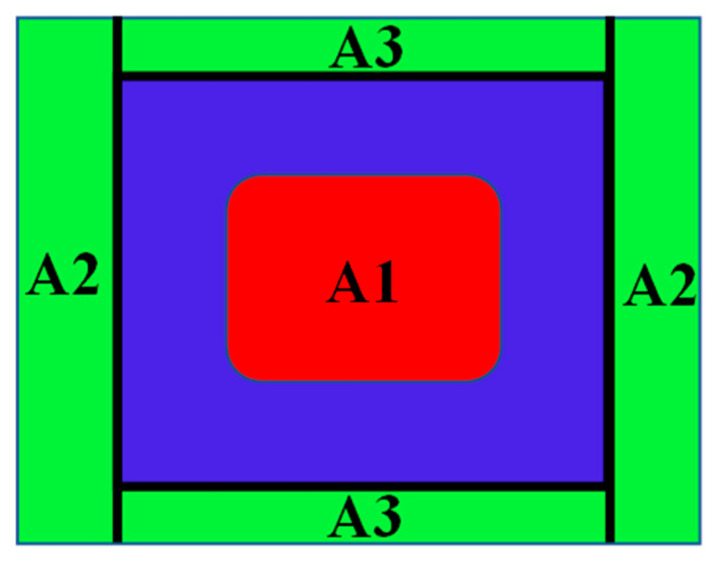
Prediction of magnetic flux density in the fluid gap.

Torque calculation begins by predicting the area affected by the magnetic field, as shown in Figure 8. Because there are several differences in magnetic flux density in the gap area, to make it easier in the analysis process, the area is divided into three, namely A1, A2, and A3. Area A1 has the most dominant magnetic flux density compared to areas A2 and A3. To determine the magnetic flux density in the fluid gap, the number of turns of the coil wire, the yield stress ($\tau_{y}$) and the relative permeability of MRF-132 DG need to be determined. Referring to the calculations conducted by Saini et al. [12], the number of turns that can fill the coil space can be determined by Equation (20). When affected by a magnetic field, yield stress (kPa) can be calculated by the polynomial Equation (21). Finally, the MRF permeability value can be calculated by Equation (22).

(20) $$N=\frac{2p_{f}\left(2l_{cs}w_{c}\right)}{\pi d_{c}{}^{2}}$$

where N is the number of turns, $p_{f}$ is the packing factor assuming the value is 0.6 for AWG 30 wire [37], and $d_{c}$ is the diameter of the coil wire. Therefore, with a current variation of 0.5 A, 1 A, 1.5 A, and 2 A, it can be converted into turn-ampere parameters of 150 tA, 300 tA, 450 tA, and 600 tA.

(21) $$\tau_{y}\left(B\right)=52.962B^{4}-176.51B^{3}+158.79B^{2}+13.708B+0.1442$$

(22) $$\mu =\frac{B}{H}$$

where *B* is the magnetic flux density, and *H* is the magnetic field intensity.

**Figure 9:**
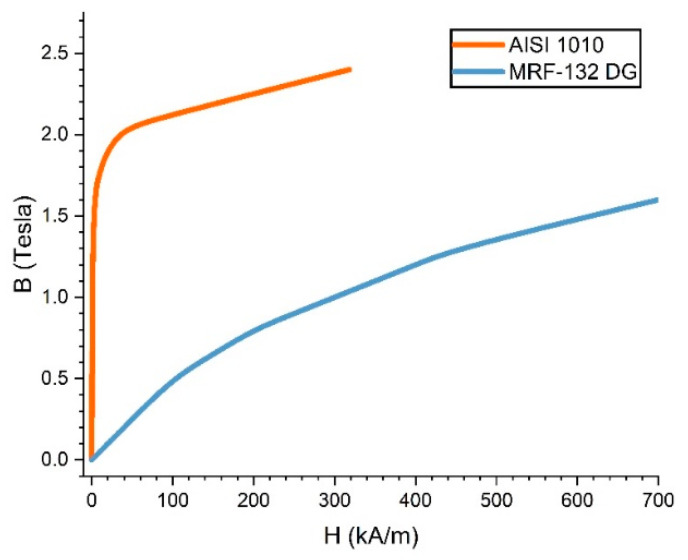
B-H curves of MRF-132 DG [34] and AISI 1010 [33].

Relative permeability is an essential factor affecting the saturation properties of a magnet. Every ferromagnetic material has a different response when exposed to a magnetic field. The B-H curve generally describes the difference. Figure 9 shows the B-H curve of the MRF-132 DG and AISI 1010 materials. MRF-132 DG has a curve-fit shape that shows that MRF’s relative permeability is non-linear. Meanwhile, AISI 1010 shows a characteristic where the magnetic flux density is more significant with the same magnetic strength than MRF with quite extreme saturation at a large magnetic flux density.

**Figure 10:**
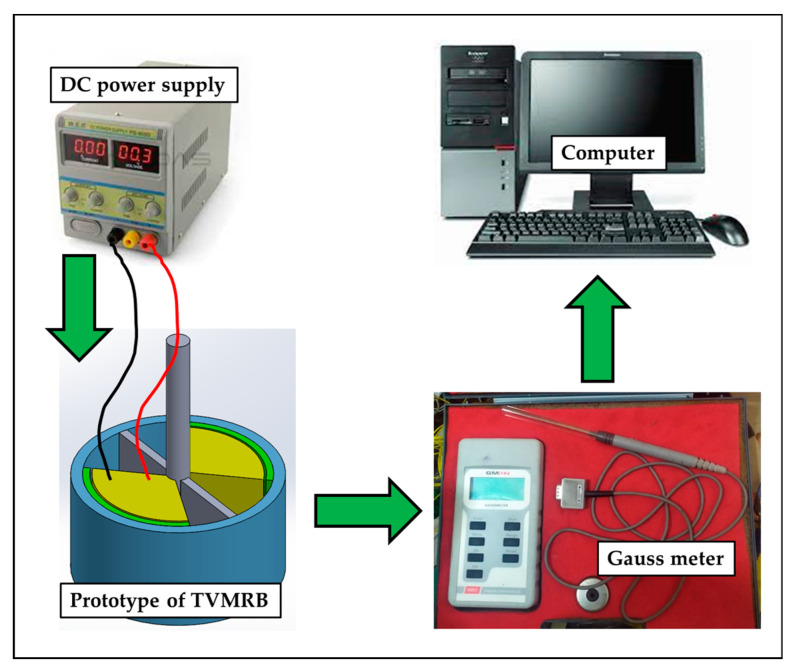
Schematic of magnetic flux density testing on the TVMRB prototype.

MRF and AISI 1010 have non-linear permeability; therefore, several calculation scenarios are made to make it easier to predict the magnetic flux density in this study. First, it is calculated theoretically with an air-filled fluid gap. Second, it is determined by computational simulation with an air-filled fluid gap. Third, it was carried out experimentally with the scheme, as shown in Figure 10. For example, the prototype was made with a fluid gap of 1 mm. Fourth, it is calculated theoretically with a fluid gap containing MRF, but the relative permeability value is kept constant at 2.78 following the research conducted by Saini et al. [12]. Fifth, the simulation is carried out with a fluid gap containing MRF. Then, to simplify the analysis, the first to fifth scenarios are carried out in area A. Finally, the total torque of TVMRB is calculated assuming a constant rotor speed of 10 rpm and by taking the magnetic flux density values in a simulation in areas A1, A2, and A3.

## 3. Results and Discussion

### 3.1. Torque Characteristics

**Figure 11:**
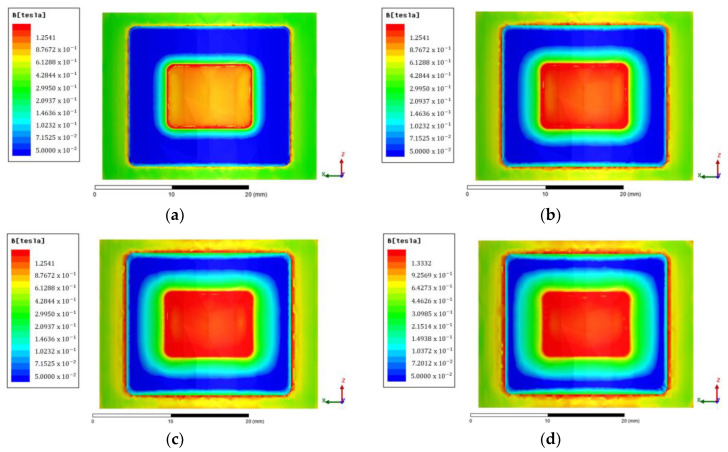
Contour of magnetic flux density at 0.5 mm fluid gap with variations of: (**a**) 150 tA; (**b**) 300 tA; (**c**) 450 tA; (**d**) 600 tA.

Figure 11 shows the magnetic flux density contour in the fluid gap area. The red to blue colours indicate the strength level of the magnetic flux effect. In this study, the magnetic flux parallel to the coil is considered zero because its value is relatively small. Although the value of magnetic flux density can be known by simulation, there is still a need for theoretical and experimental validation.

**Figure 12:**
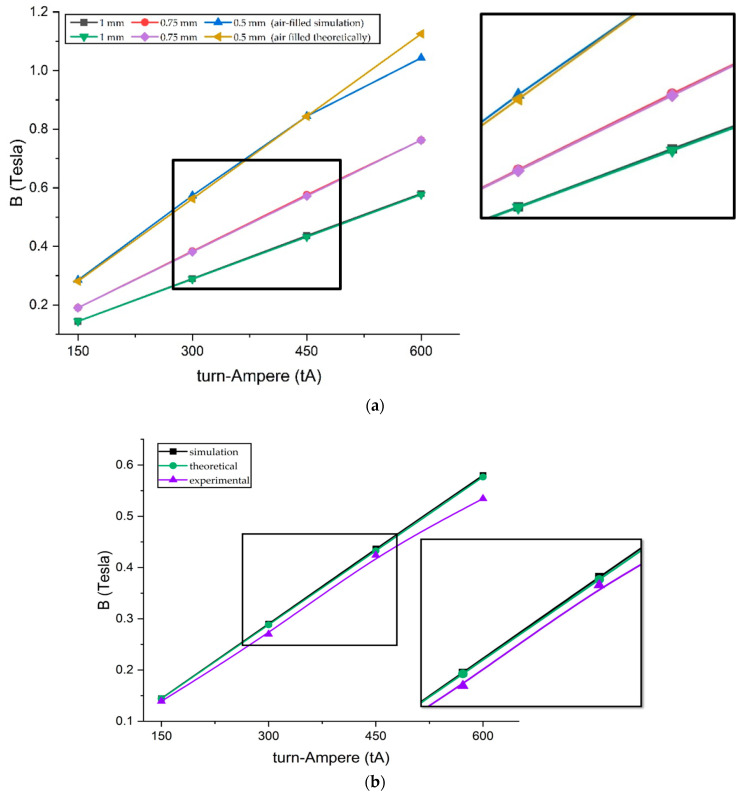
Result of validation tests: (**a**) First scenario vs second scenario; (**b**) First, second, and third scenarios at 1 mm fluid gap.

Figure 12a is a comparison chart between the first and second scenarios. The results show that the value of magnetic flux density between theoretical and simulation tests on fluid gaps filled with air is almost the same. A significant error occurs when the magnetic flux density reaches more than 0.8 Tesla. However, this is normal because the permeability of the AISI 1010 material during theoretical testing is considered constant. In contrast to the simulation, which places the AISI 1010 permeability parameter according to Figure 9, there is a saturation level of the material when the magnetic flux density value is high, whereas Figure 12b is a comparison of the first, second, and third scenarios with a fluid gap of 1 mm. Experimental test results are lower than theoretical and simulation, but the error is low, not more than 9%. Therefore, the theoretical, simulation, and experimental analysis in this study can be said to be valid.

**Figure 13:**
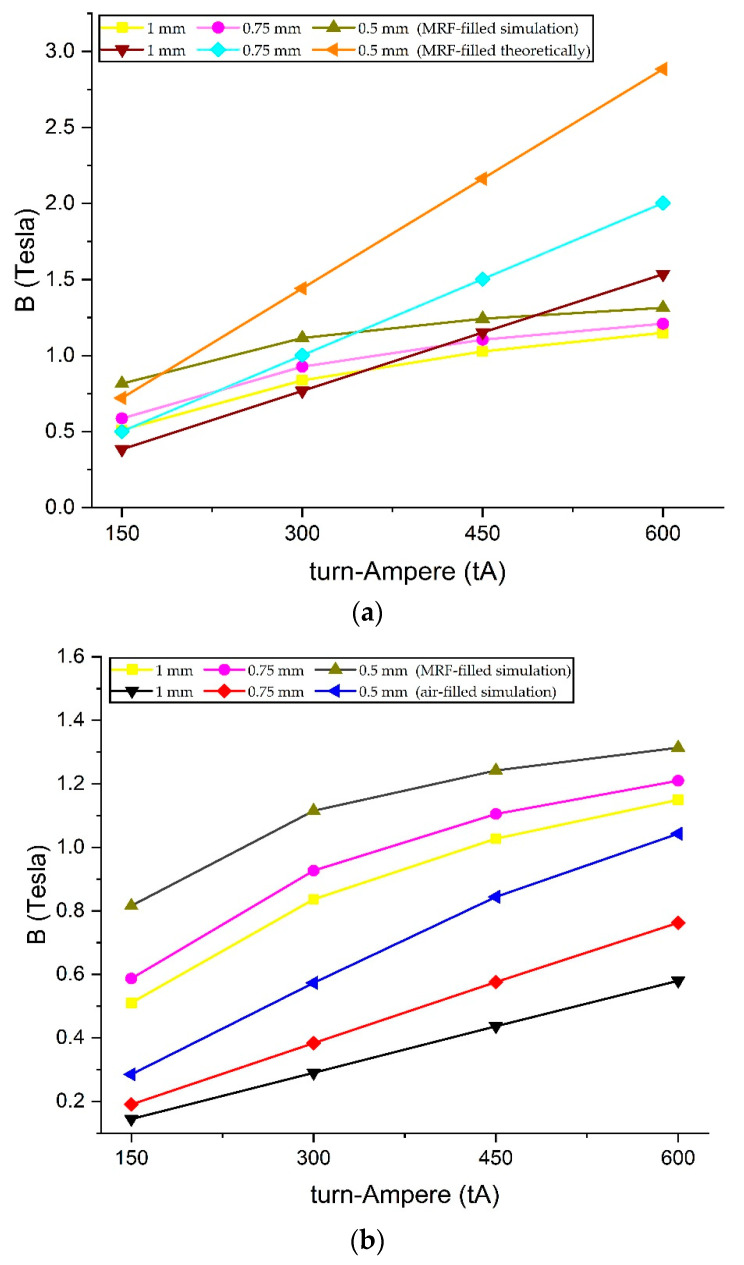
Comparison of magnetic flux density: (**a**) Fourth scenario vs fifth scenario; (**b**) Second scenario vs fifth scenario.

After the validity, the fourth and fifth scenarios can be carried out by inserting MRF material into the fluid gap. Figure 13a shows a graph difference between the fourth and fifth scenarios. The difference becomes significant as the magnetic flux density increases. However, this happens because, when theoretical, the relative permeability of the MRF was kept constant at 2.78, referring to the research made by Saini et al. [12]. In contrast, the MRF permeability during the simulation is inputted according to Figure 9. From these results, it can be stated that the theoretical method has limitations regarding the permeability parameters for calculating magnetic flux density involving MRF. It requires complex calculations to complete. Therefore, for calculating TVMRB torque, the value of magnetic flux density is taken from the simulation results. Not only in areas A1 but also areas A2 and A3, even though the magnetic flux density value is low whereas Figure 13b is a comparison graph between the second and fifth scenarios. The results show that by simulation, the value of magnetic flux density in the fluid gap containing MRF is more significant than in the fluid gap containing air because MRF has a greater permeability. However, the significant increase is only at 300 tA, considering that the MRF permeability is not linear.

**Figure 14:**
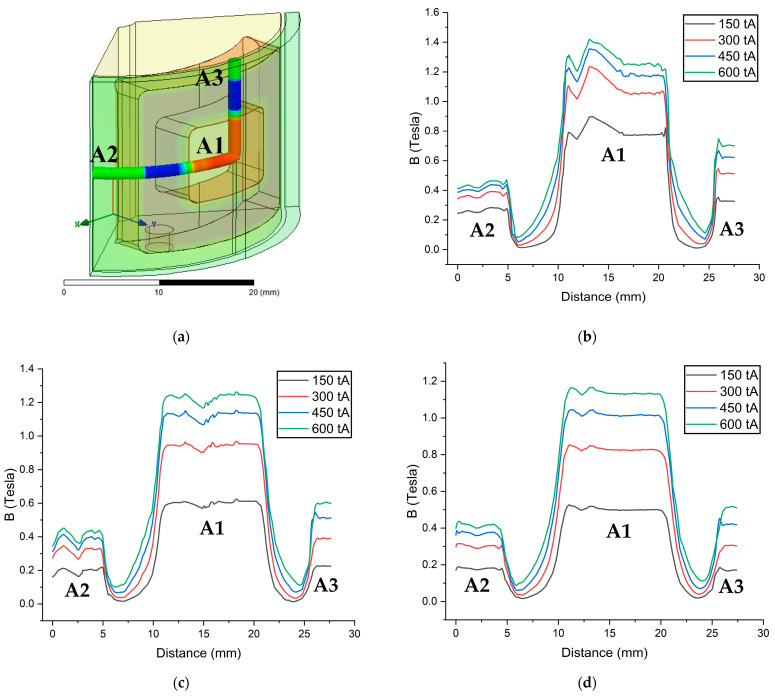
Simulation results of magnetic flux density in areas A1, A2, and A3: (**a**) Test path scheme; (**b**) Testing with a fluid gap of 0.5 mm; (**c**) Testing with a fluid gap of 0.75 mm; (**d**) Testing with a fluid gap of 1 mm.

TVMRB torque is determined from the resulting values $\Delta P_{off-state}$ and $\Delta P_{on-state}$. $\Delta P_{off-state}$ is calculated by inputting the design parameters according to Equation (3). $\Delta P_{on-state}$ is obtained from the magnetic flux density value conversion according to Equation (4). Figure 14 shows the value of the magnetic flux density in areas A1, A2, and A3. Because $\Delta P_{on-state}$ in each area is different, in determining the total, a relationship between the areas is made with Equation (23).

(23) $$\Delta P_{on-state}\left(total\right)={\left(\frac{2}{\Delta P_{A3}}+\frac{1}{\Delta P_{A1}}\right)}^{-1}+2\Delta P_{A2}$$

**Figure 15:**
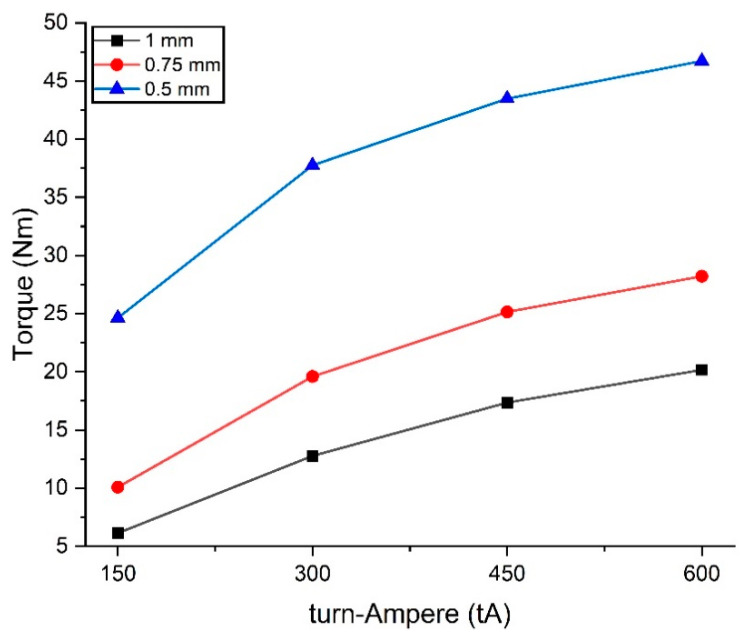
TVMRB torque achievement in each variation.

Figure 15 shows the TVMRB torque at each turn-ampere and fluid gap variation. The torque value will increase with the increase in turn-ampere, but the torque will decrease if the fluid gap is enlarged. This happens because of the influence of the magnetic flux density. When viewed from the turn-ampere variation, the most significant increase in torque occurs at 300 tA. After that, the increase in torque tends to be sloping because of the influence of the magnetic saturation of the MRF material. In terms of the fluid gap, the torque increases significantly, with a fluid gap of 0.5 mm.

The TVMRB design is targeted to have a minimum torque of 10 Nm. From the calculation results, the torque generated in most variations can exceed the target. There is only one condition where the torque is below. When the turn-ampere is at 150 tA with a fluid gap of 1 mm, the resulting torque is 6.14 Nm, whereas the highest torque value is 46.71 Nm with a variation of 600 tA and a fluid gap of 0.5 mm.

Torque achievement is indeed one of the critical factors in the TVMRB design. However, according to Imaduddin et al. [35], there are several other factors, such as operation range (λ) and power consumption, to determine brake performance. Valves must have a large $\Delta P_{on-state}$, so the torque generated is also significant. On the other hand, the valve is expected to have a low $\Delta P_{off-state}$ value so that during the off-state condition, there is no torque generated so as not to reduce the range of movement of the rotor. Then, the comparison between $\Delta P_{on-state}$ and $\Delta P_{off-state}$ is called the operational range, which can be calculated by Equation (24). The operational range is said to be high if $\Delta P_{on-state}$ is high and $\Delta P_{off-state}$ is low, and vice versa.

(24) $$\lambda =\frac{\Delta P_{on-state}\;}{\Delta P_{off-state}}$$

**Figure 16:**
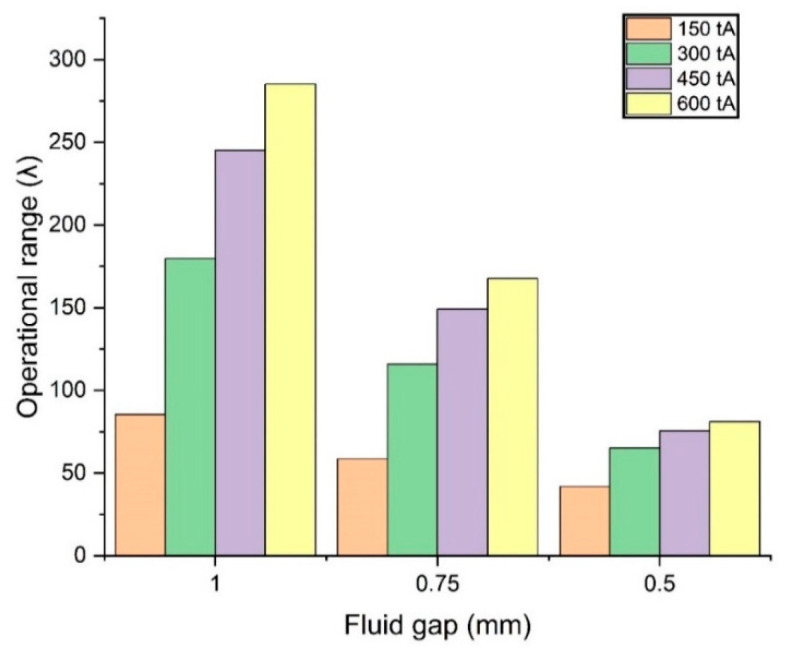
Fluid gap and turn-ampere relationship to operation range.

Figure 16 is the result of calculating the operational range of the TVMRB design. The operational range increases with increasing turn-ampere and fluid gap. The increase occurred significantly in the valve gap of 1 mm at each turn-ampere variation. When looking at the torque calculations, variations in the fluid gap of 1 mm produce lower torque than the fluid gaps of 0.5 mm and 0.75 mm. However, because of the off-state condition, the fluid gap variation of 1 mm produces a low $\Delta P_{off-state}$ and the operational range can increase significantly.

**Figure 17:**
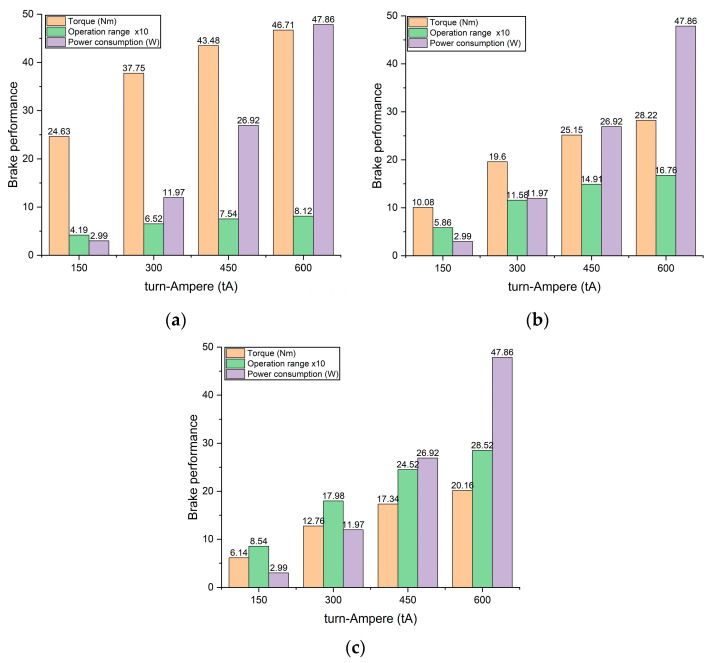
Brake performance results with fluid gap of: (**a**) 0.5 mm; (**b**) 0.75 mm; (**c**) 1 mm.

Power consumption determines brake performance. In this study, the power consumption is calculated based on the current and coil resistance value. The calculation results show that the lowest power consumption is 2.99 W at 150 tA, and the highest value is 47.86 at 600 tA. Finally, the comparison between torque, operating range, and power consumption generated by TVMRB is presented in Figure 17.

### 3.2. House of Quality (HOQ)

**Figure 18:**
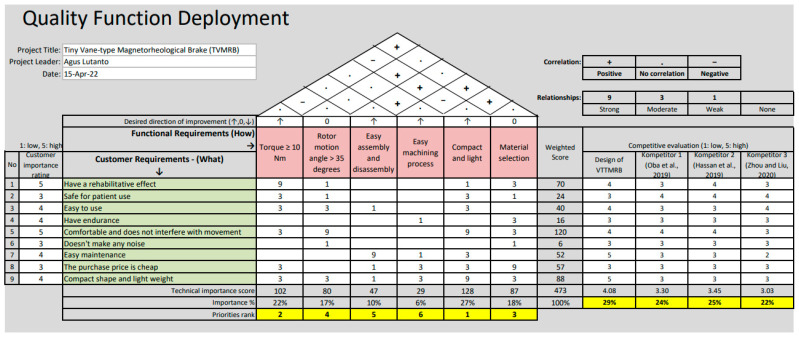
HOQ of the TVMRB design.

HOQ is a tool that connects consumer desires with the technical steps of making designs [38]. Referring to Figure 18, two main priorities emphasize making the TVMRB design. First, the torque generated, and second, designs must be compact and light. From the results shown in Figure 15, the torque has met the criteria because by testing several variations, the torque produced can reach more than 10 Nm, whereas in terms of compact and light, the TVMRB design has a tiny size with a diameter and height of 45 mm and 28 mm, respectively, smaller than the design made by Kikuchi et al. [22]. For weight, the design was simulated using the Solidwork application, with a result of 163.72 g. Then, the TVMRB design is compared with the other three competitors and assessed in terms of meeting consumer needs, which achieved the first rank with an average value of 4078.

## 4. Conclusions

The application of QFD in making an MRB design needs to be considered. With this method, the direction of design development becomes more measurable so that the product will follow customer needs with clear steps. The specified QFD successfully made the TVMRB design, starting from the fulfillment of the design criteria. At the end of the article, the HOQ shows the value of the TVMRB design compared to other designs. The ranking value is based on nine customer requirement items linked to six technical/functional requirements. From the analysis result, TVMRB achieves the first rank, with development priorities focused on torque and compactness.

In terms of design, TVMRB is a simple design, is easy to manufacture, and it is easy to remove and install each part. For applications on AFO devices, the TVMRB has an angle of movement of 70 degrees, exceeding the angle of the ankle when walking (35 degrees), making it more flexible. The design also has a compact size and is lightweight. Diameter, height, and weight are 45 mm, 28 mm, and 163.72 g, respectively.

TVMRB is targeted to produce a minimum torque of 10 Nm in order to have a rehabilitation effect. The simulation test results show that the torque produced can reach more than the target. However, to choose the best parameters, it is not only torque but other factors that affect brake performance. By analyzing the results of torque, operational range, and power consumption, there are two options for choosing variations. In the first option, with a fluid gap of 0.75 mm and a turn-ampere of 150 tA (current 0.5 A), the resulting torque is 10.08 Nm. The second option is a fluid gap of 1 mm and a turn-ampere of 300 tA (1A current); the resulting torque is 12.76 Nm. These two options were chosen because if the fluid gap is reduced to 0.5 mm, the torque will increase, but the operational range will be low. Meanwhile, if the current is increased by more than 300 tA, the torque and operational range will increase, but in terms of power consumption, it will also increase and can potentially cause heat to the coil. However, this study does not discuss how much influence the turn-ampere has on the heat in the coil and its effect on the performance of the TVMRB; therefore, it becomes an opportunity for further research. Moreover, further experimental work comprising prototype development and its characterization will be the top priority in future work. In addition, several application devices using the proposed tiny controllable MRB such as ankle foot orthosis (AFO) and auto-door closure of vehicles can be manufactured and tested in the near future.

## Figures and Tables

**Table 1:** Characteristics of MRF-132 DG [34].

Properties	Value/Limits
Codes of MR fluids	MRF-132 DG
Based fluid	Hydrocarbon
Appearance	Dark gray liquid
Viscosity @40 °C	0.112 ± 0.02 Pa-s
Density range	2.95 to 3.15 g/cm^3^
Work temperature	−40 to 130 °C
Solid content by weight	80.98%
Flash point	>150 °C

**Table 2:** TVMRB component materials.

Parts	Types	Materials
Cover	Non-ferromagnetic	Aluminium
O-ring	Non-ferromagnetic	Rubber
Rotor	Non-ferromagnetic	Aluminium
Housing	Non-ferromagnetic	Aluminium
Inner valve	Ferromagnetic	Steel AISI 1010
Outer valve	Ferromagnetic	Steel AISI 1010
Connecting circlip	Non-ferromagnetic	Stainless steel
Coil	Non-ferromagnetic	Copper

**Table 3:** Complete parameters of static valve design.

Parameters	Descriptions	Units	Value
$\mu_{st}$	Magnetic permeability of AISI 1010	H/m	0.000838694
$\mu_{a}$	Magnetic permeability of air	H/m	0.000001256
$\mu_{MRF}$	Magnetic permeability of MRF	H/m	Not-constant
ω	Angular velocity (10 rpm)	rad/s	1.0467
$l_{o}$	Outer valve length	mm	34.98
$l_{i}$ = L	Inner valve length = Fluid gap length	mm	32.58
$l_{c}$	Coil core length	mm	11.5
$l_{cs}$	Coil space length	mm	5
$l_{ca}$	Coil chord to coil core length	mm	3.49
$w_{o}$	Outer valve width	mm	1.5
$w_{c}$	Coil core width	mm	5
$w_{gap}$	Fluid gap width	mm	0.5, 0.75, 1
$h_{v}$ = $h_{bl}$	Valve height = Rotor blade height	mm	22
$h_{c}$	Coil core height	mm	8.5
$r_{i}$	Inner valve radius	mm	18
$r_{s}$	Rotor shaft radius	mm	2.5
$r_{bl}$	Rotor blade radius	mm	20 (on w_gap_ 0.5)
α	Valve angle	°	110

## Data Availability

Not applicable.
